# Complete metastasectomy in renal cell carcinoma: a propensity-score matched by the International Metastatic RCC Database Consortium prognostic model

**DOI:** 10.3332/ecancer.2019.967

**Published:** 2019-10-14

**Authors:** Aline F Fares, Daniel V Araujo, Vinicius Calsavara, Augusto Obuti Saito, Maria Nirvana Formiga, Aldo A Dettino, Stenio Zequi, Walter H da Costa, Isabela W Cunha

**Affiliations:** 1 Department of Medical Oncology, AC Camargo Cancer Center, São Paulo 01525-001, Brazil; 2 Department of Medical Oncology, University Health Network, Toronto, ON M5G 1L7, Canada; 3 Department of Epidemiology and Statistics, AC Camargo Cancer Center, São Paulo 01525-001, Brazil; 4 Department of Urology, AC Camargo Cancer Center, São Paulo 01525-001, Brazil; 5 Department of Urology, Santa Casa de Misericordia de São Paulo, São Paulo 01525-001, Brazil; 6 Department of Pathology, AC Camargo Cancer Center, São Paulo 01525-001, Brazil; 7 Department of Pathology, Rede D’OR-Sao Luis, São Paulo 01525-001, Brazil; * Aline F Fares and Daniel V Araujo contributed equally to writing this article.

**Keywords:** metastasectomy, clear cell renal cell carcinoma, targeted therapy, propensity score matching

## Abstract

**Introduction:**

We evaluated overall survival (OS) benefit of complete metastasectomy (CM) in metastatic renal cell carcinoma (mRCC) using a propensity score-matched (PSM) analysis to balance groups by age, gender and by the International Metastatic RCC Database Consortium prognostic model (IMDC).

**Methods:**

We included patients (pts) treated at the AC Camargo Cancer Center between 2007 and 2016. Pairs were matched by age, gender and IMDC. Kaplan–Meier survival estimates and Cox proportional hazard models were used to evaluate OS on CM and no-CM group.

**Results:**

We found 116 pts with clear cell mRCC. After PSM, the number was reduced to 74 (37 CM, 37 no-CM). The median OS for CM and no-CM was 98.3 months and 40.5 months, respectively (hazard ratio 0.24 95%CI 0.11–0.53 p < 0.001). The OS benefit of CM was confirmed on favourable and intermediate IMDC but was absent on poor IMDC. The CM group received less systemic therapy than the no-CM group. Ten pts in the CM group still have no evidence of disease (NED).

**Conclusion:**

After matching for age, gender and IMDC, we found CM impacts on OS and also diminishes the need for systemic treatment. Survival benefit was confirmed for favourable/intermediate IMDC but not for the poor IMDC prognostic model. Further studies correlating IMDC and metastasectomy are needed to guide clinical decision-making.

## Introduction

The treatment of metastatic renal cell carcinoma (mRCC) has changed dramatically over the last 30 years. Following an evolution from interleukin and interferon to tyrosine kinase inhibitors (TKI) and immunotherapy, survival rates have risen significantly [1]. Nevertheless, cure with systemic treatments alone is elusive for the vast majority of patients, and local treatments remain an important treatment pillar often pursued whenever possible.

Oligometastatic RCC can be treated radically as a localised disease [2]. Surgery or stereotactic radiosurgery of metastatic sites can be offered aiming to prolong time-to-treatment and OS [2, 3]. Usually, these treatments are offered to a selected population with good performance status and fit enough to undergo surgical interventions with an underlying active cancer. Prior reports, mostly retrospective, found improvements in survival, showing 5-year survival rates of up to 80% in patients undergoing metastasectomy, with variations depending on site of metastasectomy [4–9]. Lung and pancreatic metastasectomies appear to have higher rates of success and better survival, whereas bone and liver are related to worse survival [10, 11].

Although these studies report impressive survival numbers, the question of whether metastasectomy per se is directly associated with survival or if disease biology of these indolent and slow progressor tumours is the main factor, is yet to be answered. Verbiest *et al* [12] evaluated if the molecular subtypes ccRCC1–4 are prognostic in the setting of CM. They studied 43 clear cell RCC (ccRCC) patients and found subtype-dependent impact in terms of disease relapse after CM: ccRCC1&4 have 5-year disease-free survival (DFS) of 10%, whereas ccRCC2&3 have 5-year DFS of 27%. Certainly, this supports the importance of tumour biology underlying metastasectomy benefit and suggests that ccRCC1&4 might be better treated with systemic treatment alone. However, these subtypes are obtained by RNA-sequencing, a resource not readily available, and that can bring financial challenges to clinical practice.

A promptly available method to evaluate prognostic in mRCC is the International Metastatic Renal Cell Carcinoma Consortium (IMDC) risk factor [13]. It includes clinicolaboratorial data that categorise patients in favourable/intermediate/poor prognosis and is widely used in clinical trials and clinical practice to advise therapeutical guidelines. Cytoreductive nephrectomy (CN) was evaluated in a large cohort of synchronous metastatic patients and was found to impact on better survival for patients with 0–3 IMDC criteria; those with 4–6 IMDC criteria do not derive survival benefit from CN [14]. IMDC is also used for treatment decision-making in first-line systemic treatment for mRCC and as of today, first-line treatment as per guidelines is nivolumab and ipilimumab for intermediate/poor prognosis and TKI for favourable prognosis [15, 16].

Despite the clear importance of IMDC criteria as a clinical tool for decision-making, to the best of our knowledge, there are no reports evaluating the role of metastasectomy stratified per IMDC prognostic groups. In this study, we investigate the role of metastasectomy in a PSM population accounting for known prognostic factors in mRCC such as age, gender and IMDC criteria.

## Materials and methods

The present study was conducted after approval of the AC Camargo Cancer Center institutional ethics committee. Using the ACC biobank, we searched for metastatic tissue samples in RCC. We included patients with clear cell histology and absence of sarcomatoid or rhabdoid features who underwent previous nephrectomy. During radical nephrectomy, retroperitoneal lymphadenectomy was restricted to the renal hilum and was performed for staging purposes only. In nephron-sparing procedures, lymph node dissection was not performed. We included patients who underwent CM and also patients treated without metastasectomy and with targeted therapy (TT) alone who fit inclusion criteria.

Complete metastasectomy (CM) was defined as no evidence of residual disease following surgery, neither on index organ nor other organs and therefore considered a curative intent surgery. The decision to perform CM was at the discretion of the treating physician and patient acceptance. CM was generally advised for patients with limited resectable disease and low cardiovascular surgery risk. The surgery could have been done at any time during the treatment course. Metastases locations sites were classified as: lungs, bones, liver, central nervous system (CNS), lymph nodes, intra-abdominal (including pancreas, adrenal, abdominal lymph nodes) and skin and subcutaneous tissue.

Clinicopathological collected information were: age, gender, Fuhrman/International Society of Urological Pathology (ISUP) grade, site of CM, date of first diagnosis, date of recurrence, status and date of the last follow-up. Karnofsky performance scale (KPS), calcium, neutrophils and platelets values, as well as time from the first diagnosis to metastasis, were annotated as independent variables and as part of the IMDC risk criteria. Baseline blood analysis was collected at the time of metastases diagnosis. Patients were then categorised according to IMDC prognosis criteria into good, intermediate or poor prognosis [18]. Staging was assigned using the 2010 American Joint Committee on Cancer (AJCC) classification.

We defined synchronous metastasis as patients who had distant metastasis diagnosed upfront or within 6 months from the first diagnosis. Recurrence was defined as a new site of a metastatic disease diagnosed clinically, by imaging or biopsy.

All patients underwent a metastatic workup before metastasectomy, to rule out other sites of metastases. This included CT of the chest, abdomen and pelvis in all patients; bone scan and brain MRI if clinically advised. Systemic treatment after metastasectomy could be administered, at the discretion of the treating physician. Patient information was obtained from the AC Camargo’s electronic medical chart. Surveillance after CM typically consisted of CT scan of the chest, abdomen and pelvis every 4 months for the first 2 years and every 6 months consequently, as per institutional policy.

### Statistical analysis

We matched and balanced CM/no-CM in pairs for age, gender and IMDC risk factor. Matching and balancing of the empirical distributions were done using the MatchIt package in R, version 3.1.1 (https://www.r-project.org/). This propensity score analysis helps to make CM/no-CM homogeneous in terms of age, gender and IMDC risk factor. Hence, it provides less bias in the evaluation of metastasectomy impact on OS.

Association between clinicopathological variables and CM status were performed by chi-square and Fisher’s exact test, as adequate. Kaplan–Meier curves were used to predict OS and the curves were compared using log-rank test. Cox proportional hazards regression model was used to assess the prognostic significance of CM in univariable and multivariable analysis, adjusting for CNS metastasis, bone metastasis, intra-abdominal metastasis and IMDC criteria. These variables were selected based on statistical significance in the univariable model. *p* values are derived from two-tailed tests. All analyses were performed using SPSS v24 and software R version 3.5.

## Results

### Patients demographics

Of all patients diagnosed with mRCC between 2007 and 2016, 116 matched study inclusion criteria. 70% were male and the median age was 57 years (32–82). Synchronous metastasis was present in 41 patients (35%).

**Figure 1.**
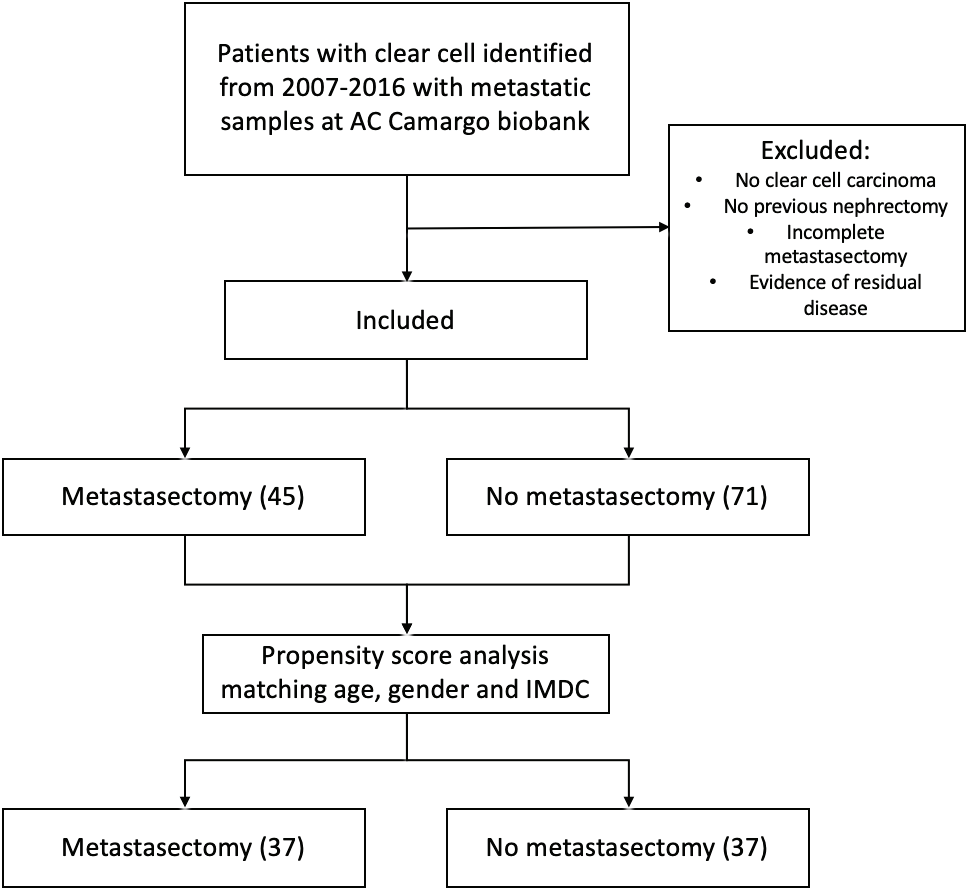
Exclusion process flow chart and propensity score analysis.

After matching pairs for age, gender and IMDC criteria, groups were reduced to 74 patients: 37 on the CM group and 37 on the no-CM group as depicted in the flowchart (Figure 1). From the cohort of paired patients, 21 patients (28%) had synchronous metastasis; 53 (72%) patients had localised disease at the time of diagnosis and developed further metastases 6 months or more after the first diagnosis of RCC.

After the matched paired analysis, clinical and prognostic characteristics were well balanced between CM and no-CM groups as shown in Table 1. There was no association between IMDC criteria and CM (*p* = 1.0). Twenty-one patients (28%) were classified as IMDC good prognosis, 49 (66%) were intermediate and 4 (5%) were poor prognosis. The most common site of metastasis was lung (86%), followed by bones (50%), lymph nodes (39%), intra-abdominal metastasis (30%), liver (22%), CNS (22%) and skin and subcutaneous (18%), as shown in Table 2. The most common sites of metastasectomy were lungs (84%), bones (49%), intra-abdominal (32%), lymph nodes (30%), liver (27%), skin and subcutaneous (16%) and CNS (13%). From all 34 patients in the CM group, 20 (59%) underwent one session of metastasectomy, 11 (32%) received two sessions and 6 (18%) received three or four sessions of metastasectomy. CNS was the only site of metastasis where we found inverse correlation with metastasectomy, likely because patients with CNS metastasis are usually not in a good performance to undergo metastasectomy or because it is a technically challenging site to perform CM (Table 2). Others sites of metastasis had no correlation with metastasectomy.

From all 74 patients, 62 patients (84%) received TT at some point of their disease course. Of note, all 12 (16%) who never received systemic therapy were in the CM group: 4 (33%) never recurred and have NED; 5 (42%) had recurrence and underwent a second metastasectomy and are now on NED status; 1 (8%) underwent two additional metastasectomies and is now on NED status as well; the other 2 (16%) recurred and did not receive further treatment due to low KPS or patient preference.

With respect to the type of systemic therapy, 43 patients (69%) received sunitinib as first-line therapy, 17 patients (27%) received pazopanib and 3 (5%) received temsirolimus. Table 3 summarises types of TT for systemic treatment per IMDC criteria.

Of note, from the full cohort of 74, only 40 (54%) initiated the second line of systemic therapy and from these, 28 (70%) were in the No-CM cohort, whereas only 12 (30%) of the CM cohort received the second line of systemic treatment. 12 patients (30%) from the CM group and 28 patients (70%) from the no-CM group, as shown in Table 4. Of these, 14 patients (35%) received everolimus, 7 patients (17.5%) received pazopanib and the others received other TT, immunotherapy or even chemotherapy.

### Survival outcomes

After a median follow-up of 70.4 months, median OS for the paired-up population was 78 months. At the time of analysis, 35 patients (46.7%) had died: 16 patients (47%) from the CM group and 19 (56%) patients from the no-CM group.

**Figure 2.**
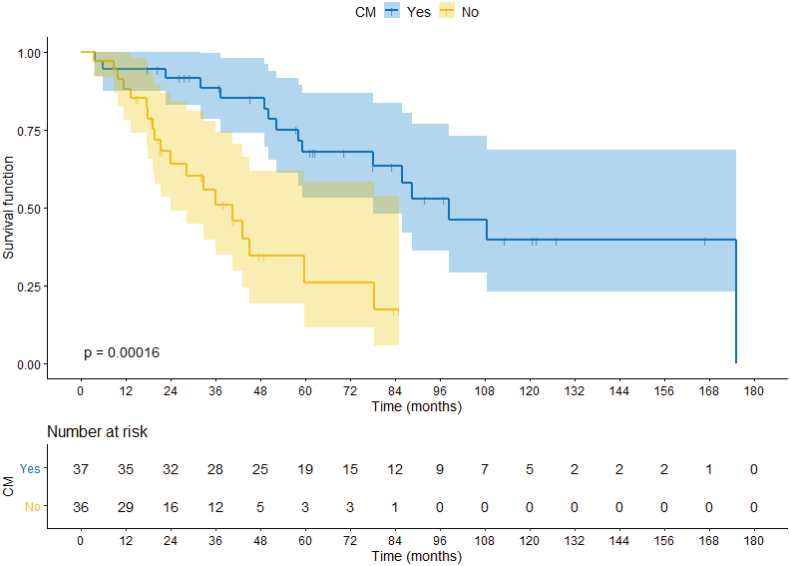
OS comparing CM group with no-CM group.

Comparing the CM group with the no-CM group, median OS was 98.3 months versus 40.5 months (hazard ratio (HR) 0.24 95%CI 0.11–0.53 *p* < 0.0001) respectively, as shown in Figure 2. When the cohort is segregated into IMDC risk groups, there was a significant survival difference between favourable prognosis versus intermediate versus poor prognosis (88.05 months, 59.72 months and 18.03 months, respectively; HR 0.15 95%CI 0.04–0.52 *p* = 0.002). There were no significant statistical differences between synchronous or metachronous metastases, probably because groups were paired according to time from diagnosis to treatment less than 1 year, one of the IMDC criteria (HR 1.01 95%CI 0.69–1.46 *p* = 0.96).

**Figure 3.**
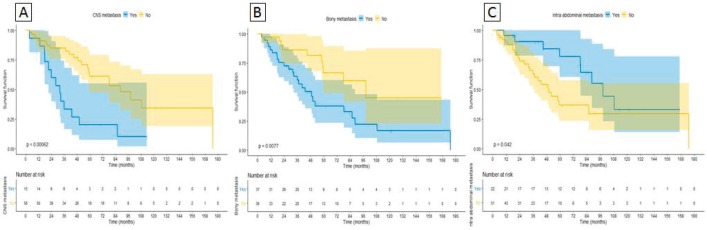
Sites of metastasis correlated with OS. (A): CNS metastasis. (B): Bony metastasis. (C): Intra-abdominal metastasis.

On univariable analysis, CNS and bone metastases were associated with worse OS, whereas intra-abdominal metastases were associated with improved OS (HR 3.17 95%CI 1.58–6.38 *p* = 0.001, HR 1.62 95%CI 1.21–2.35* p* = 0.01 and HR 0.67 95%CI 0.45–0.99* p* = 0.047, respectively) as depicted in Figure 3A, 3B and 3C. On multivariable analysis, even though IMDC criteria had a trend towards statistical significance (HR 0.29 95%CI 0.07– 1.1* p* = 0.08), CM was the only significant variable (HR 0.21 95%CI 0.09– 0.49* p* < 0.0001), after adjusting for IMDC, bone metastasis, CNS metastasis and intra-abdominal metastasis (Table 5).

We also analysed the impact of CM on OS for good/intermediate and for poor IMDC prognosis group separately. The benefit was confirmed for the first group, (HR 0.22 95%CI 0.09–0.5* p* < 0.0001). However, for the poor prognosis group, there was no benefit added by CM (HR 1.61 95%CI 0.14–18.31* p* = 0.69).

## Discussion

In the present study, we found that complete surgical resection of metastasis in clear cell RCC significantly improves OS when compared to systemic treatment alone. This benefit was restricted to IMDC good/intermediate prognosis patients and was not present in the poor prognosis group. We also found that patients undergoing CM often use less systemic therapy than patients on the no-CM group, suggesting that CM prolongs time-to-treatment-initiation.

Our results are in agreement with prior retrospective studies, supporting survival advantage for patients undergoing metastasectomy [4–9]. However, this is the first study to report results on a homogeneous cohort of patients treated with TT and balanced by IMDC criteria.

Even though no randomised clinical trials have evaluated the role of complete surgical metastasectomy (CM) in mRCC, observational studies—including case series, cohort studies and meta-analysis—have shown important survival benefit in favour of aggressive treatment of oligometastatic RCC. These previous reports were mostly conducted in a time when interleukin-2 and interferon were the standard systemic treatments and few patients had been treated with TT. Alt *et al* [17] analysed 883 patients with mRCC submitted to multiple complete surgical resections of the metastatic sites. Only 404 (45%) patients received systemic treatment and 54 (13%) received TT. They demonstrated a significant benefit in OS for pts who had CM analysing the overall cohort (4.8 years versus 1.3 years;* p* < 0.001). Jakubowski *et al* [18] evaluated patients submitted to isolated CM and found a 5 year cancer-specific survival (CSS) of 84%, but they have not reported systemic treatment used or IMDC risk factors of their population, challenging a more elaborated interpretation of results. Zaid *et al* [19] published a meta-analysis with 2,267 patients and found increased overall mortality with an HR of 2.37 (95%CI 2.03, 2.87 *p* < 0.001) for the no-CM group compared with those who underwent CM. Sun* et al* [20] recently reported a large analysis including 6,994 patients with mRCC, of which 28% underwent metastasectomy at some point of their disease. They found increase survival for the CM subgroup (HR 0.83 95%CI 0.77–0.90* p* < 0.001). However, both Zaid* et al* [19] and Sun [20]* et al* did not specify types of systemic treatment nor site of metastasectomy. They also did not have available IMDC risk factor data and is possible that groups are not well-balanced by these prognostic variables.

It remains uncertain whether this survival benefit previously demonstrated is due to surgical approach itself or to the better performance and prognosis of patients referred to surgery. Therefore, we decided to perform a matched paired analysis, balancing IMDC criteria, age and gender in both groups. This strategy reduced the number of participants, but, on the other hand, balanced the arms, reducing possible selection bias.

CM seems to only benefit patients with good or intermediate prognosis suggesting no survival benefit in poor prognosis patients. These findings need confirmation on a larger cohort, composed by a larger number of poor IMDC risk factor patients, and this has inherent difficulties, as patients with poor prognosis are usually in worse performance-status, not being offered CM.

Furthermore, herein, we discuss in details lines of treatment offered to 74 patients with mRCC, including 37 submitted to curative intent CM, and 37 who did not undergo metastasectomy and were treated with TT alone. Within the CM cohort, 10 patients (27%) are currently in NED status and have not received any systemic treatment to date. This is important as those patients who would otherwise be exposed to systemic treatments could be spared of potential serious treatment-related adverse events. This finding suggests that surgery can be a definitive treatment option to a very well-selected group of subjects.

The strength of our study lies on the homogeneous cohort of metastatic ccRCC patients, without sarcomatoid or rhabdoid features, and balanced by IMDC, age and gender. Half of the cohort underwent CM and the other half received only TT. The benefit of CM is consistent in univariable and multivariable analysis. This is the first study to allow such a detailed analysis of the population undergoing metastasectomy.

We acknowledge several limitations, including the retrospective and non-randomised nature, and small sample size. Further studies, ideally prospective and randomised, with a larger sample size able to include a reasonable number of IMDC poor-risk patients, are of great importance. The decision to treat oligometastatic patients aggressively versus standard of care systemic therapy is still not answered, especially in poor-risk patients. Our small sample size of poor-risk patients despite not allowing definitive interpretations, suggests a lack of benefit from CM, in line of what was demonstrated by Heng *et al* [13] for CN.

In summary, we have shown that CM has a significant impact on survival and remains an important treatment option in mRCC management. Our results suggest that CM benefit is restricted to good and intermediate IMDC risk patients. In addition, patients who undergo CM are less likely to require systemic treatment, suggesting a role in delaying treatment and avoiding further toxicities.

## Conclusion

Among patients with mRCC with pairs matched by IMDC, metastasectomy was associated with reduced odds of receiving systemic treatment and with a significant survival benefit. This benefit was significant mainly for favourable and intermediate-risk IMDC but was absent for the poor-risk group. Further studies with larger sample size and, in particular, including poor-risk patients are needed to guide clinical decision-making.

## Funding declaration

No financial support was necessary for this study.

## Conflicts of interest

None of the authors have any conflicts of interest.

## Figures and Tables

**Table 1.** Patients’ characteristics by CM versus no-CM.

Characteristics	Category	CM (*n* = 37)	No-CM (*n* = 37)	*p*
Age	Mean (SD)	55.92 (11%)	57.38 (11%)	0.575
Sex	Female	12 (32%)	9 (24%)	0.606
	Male	25 (68%)	28 (76%)	
Fhurman	1	2 (8%)	2 (8%)	0.879
	2	10 (42%)	10 (42%)	
	3	5 (21%)	7 (29%)	
	4	7 (29%)	5 (21%)	
IMDC criteria	Good	11 (30%)	10 (27%)	1
	Intermediate	24 (65%)	25 (68%)	
	Poor	2 (5%)	2 (5%)	
Metastasis status	Synchronous Metachronous	14 (37%) 23 (62%)	7 (19%) 30 (81%)	0.06
Systemic therapy at any time	No Yes	12 (32%) 25 (68%)	0 (0%) 37 (100%)	<0.0001
Number of metastasectomy sessions	1 2 3 or 4	20 (59%) 11 (32%) 6 (18%)	0 0 0	

**Table 2.** Metastatic sites and metastasectomy sites.

Metastasectomy site	All *N* (%)	CM *N* (%)	No-CM *N* (%)	*p*
All sites	74 (100%)	37 (100%)	37 (100%)	
Lungs	64 (86%)	31 (84%)	33 (89%)	0.36
Bones	34 (50%)	18 (49%)	19 (51%)	0.40
Liver	16 (22%)	10 (27%)	6 (16%)	0.19
CNS	16 (22%)	5 (13.5%)	11 (29%)	0.02
Lymph nodes	29 (39%)	11 (30%)	18 (48%)	0.07
Intra-abdominal	22 (30%)	12 (32%)	10 (27%)	0.50
Skin	13 (18%)	6 (16%)	7 (18%)	0.38

**Table 3.** Systemic treatment received by IMDC risk factor.

Type of systemic treatment used in first line	IMDC risk factor		
**All targeted therapy (62)**	**Favourable (21)**	**Intermediate (49)**	**Poor (4)**
Sunitinib	12 (57%)	27 (55%)	4 (100%)
Pazopanib	4 (19%)	13 (27%)	0 (0%)
Temsirolimus	1 (5%)	2 (4%)	0 (0%)
No treatment	4 (19%)	7 (14%)	0 (0%)

**Table 4.** Line of systemic therapy received in CM and no-CM groups.

Line of systemic treatment	All *N* (%)	CM *N* (%)	No-CM *N* (%)	*p*
**All lines**	**74 (100%)**	**37 (100%)**	**37 (100%)**	
First line Yes No	62 (84%) 12 (16%)	25 (68%) 12 (32%)	37 (100%) 0 (0%)	<0.0001
Second line Yes No	40 (54%) 34 (46%)	12 (32%) 25 (68%)	28 (76%) 9 (24%)	<0.0001

**Table 5.** Univariable and multivariable analysis.

Variable	Category	*N* (*n* = 74)	Death (*n* = 35)	Univariate analysis		Multivariate analysis	
				HR (95%CI)	*p*	HR	*p*
CM	CM	37	16	0.246 (0.113–0.533)	<0.0001	0.217 (0.095–0.497)	<0.0001
	No-CM	37	19				
Bone metastasis	Yes	37	25	2.638 (1.257–5.535)	0.010	1.935 (0.850–4.405)	0.116
	No	37	10	Ref			
CNS metastasis	Yes	16	13	3.176 (1.580–6.383)	0.001	2.022 (0.913–4.474)	0.083
	No	58	22	Ref			
Intra-abdominal metastasis	Yes	22	9	0.454 (0.208–0.989)	0.040	0.620 (0.243–1.585)	0.318
	No	52	26	Ref			
IMDC	Good prognosis	21	8	0.153 (0.044–0.526)	0.003	0.298 (0.077–1.158)	0.081
	Intermiate prognosis	49	23	0.184 (0.061–0.556)	0.003	0.307 (0.093–1.008)	0.052
	Poor prognosis	4	4	Red			
